# Hormetic effects of thiamethoxam on *Schizaphis graminum*: demographics and feeding behavior

**DOI:** 10.1007/s10646-024-02743-1

**Published:** 2024-03-11

**Authors:** Hina Gul, Ihsan ul Haq, Farman Ullah, Shanza Khan, Aqsa Yaseen, Kaleem Tariq, Ali Güncan, Nicolas Desneux, Xiaoxia Liu

**Affiliations:** 1 MARA Key Laboratory of Pest Monitoring and Green Management, Department of Entomology, College of Plant Protection, China Agricultural University, Beijing, 100193 China; 2 Insect Pest Management Program, Institute of Plant and Environmental Protection, National Agricultural Research Centre, Islamabad, Pakistan; 3 State Key Laboratory for Managing Biotic and Chemical Threats to the Quality and Safety of Agro-Products, Institute of Plant Protection and Microbiology, Zhejiang Academy of Agricultural Sciences, Hangzhou, 310021 China; 4 Department of Entomology, Abdul Wali Khan University Mardan, Mardan, Khyber Pakhtunkhwa Pakistan; 5 Department of Plant Protection, Faculty of Agriculture, Ordu University, 52200 Ordu, Turkey; 6 Université Côte d’Azur, INRAE, UMR ISA, 06000 Nice, France

**Keywords:** Stimulatory effects, Sublethal effects, Insecticide toxicity, Hormetic effects, Demographic parameters, Thiamethoxam

## Abstract

In agroecosystems, insects contend with chemical insecticides often encountered at sublethal concentrations. Insects’ exposure to these mild stresses may induce hormetic effects, which has consequences for managing insect pests. In this study, we used an electrical penetration graph (EPG) technique to investigate the feeding behavior and an age-stage, two-sex life table approach to estimate the sublethal effects of thiamethoxam on greenbug, *Schizaphis graminum*. The LC_5_ and LC_10_ of thiamethoxam significantly decreased longevity and fecundity of directly exposed adult aphids (F_0_). However, the adult longevity, fecundity, and reproductive days (RP_*d*_)—indicating the number of days in which the females produce offspring – in the progeny generation (F_1_) exhibited significant increase when parental aphids (F_0_) were treated with LC_5_ of the active ingredient. Subsequently, key demographic parameters such as intrinsic rate of increase (*r*) and net reproductive rate (*R*_0_) significantly increased at LC_5_ treatment. EPG recordings showed that total durations of non-probing (Np), intercellular stylet pathway (C), and salivary secretion into the sieve element (E1) were significantly increased, while mean duration of probing (Pr) and total duration of phloem sap ingestion and concurrent salivation (E2) were decreased in F_0_ adults exposed to LC_5_ and LC_10_. Interestingly, in the F_1_ generation, total duration of Np was significantly decreased while total duration of E2 was increased in LC_5_ treatment. Taken together, our results showed that an LC5 of thiamethoxam induces intergenerational hormetic effects on the demographic parameters and feeding behavior of F_1_ individuals of *S. graminum*. These findings have important implications on chemical control against *S. graminum* and highlight the need for a deeper understanding of the ecological consequences of such exposures within pest management strategies across the agricultural landscapes.

## Introduction

In agroecosystems, insects often experience lethal effects when exposed to functional doses or residues of chemical insecticides (Desneux et al. 2005; 2007). These chemical insecticides have also been reported to induce sublethal effects on exposed arthropods (Desneux et al. 2007; Ullah et al. 2019c; Gul et al. 2021; 2023). These sublethal effects can significantly impact the biological traits and population dynamics of both directly exposed insects and their descendants (Ullah et al. 2019a; Ullah et al. 2020; Shi et al. 2022; Jia et al. 2022). The occurrence of lethal or sublethal effects in insects depends on several factors with the dose or concentration of insecticide being the most crucial determinate (Cutler. 2013; Decourtye et al. 2013). Though, the dose/concentration of insecticides is the main consideration for managing the target pest, biotic and abiotic factors which can cause spatiotemporal fluctuations in concentrations are underestimated (Desneux et al. 2005). Although the sublethal concentrations/doses affect the life-history traits of exposed insects, they may also result in boosting metabolic activities and, eventually, the growth of exposed organisms is accelerated (Rix and Cutler. 2022). This biological phenomenon is termed ‘hormesis’ i.e., stimulation at low concentrations/doses and inhibition at higher concentrations/doses (Cutler et al. 2022). The hormetic effects of sublethal concentrations and dosages of pesticides should be assessed as they may inadvertently lead to increased crop injury (Guedes et al. 2016).

The greenbug, *Schizaphis graminum* (Rondani) (Hemiptera: Aphididae), is one of the most economically important pests of wheat worldwide (Hullé et al. 2020). This key pest causes direct damage through sap feeding and indirect damage by transmitting several plant pathogenic viruses, including the barley yellow dwarf mosaic virus and the sugarcane mosaic virus (Hullé et al. 2020). Despite several options for pest management in general (Wang et al. 2021; Zhang et al.^ 2021^; Nieri et al. 2022; Ullah et al. 2022), insecticide application remains an important tool that farmers easily use (Desneux et al. 2022; Kenis et al. 2023). Thiamethoxam is a neonicotinoid insecticide commonly used to control sap-sucking insect pests in various crops (Ullah et al. 2020; Zhang et al. 2022). This insecticide specifically binds to nicotinic acetylcholine receptors (nAChRs) in insect nervous systems, generating nerve stimulation, paralysis, and death (Tomizawa and Casida. 2005). Apart from lethal effects, insecticides, especially neonicotinoids, have sublethal effects on arthropod’s physiological and behavioral characteristics, such as lifespan, developmental period, fecundity, host finding, and feeding activity (Ullah et al. 2019b; Aeinehchi et al. 2021; Hafeez et al. 2022). These effects can be intergenerational, influencing offspring indirectly (Shi et al. 2022), resulting in changing communities and ecological services (Lu et al. 2012; Abd Allah et al. 2019). Hormetic effects caused by insecticides have recently been reported in melon aphids *Aphis gossypii* Glover (Hemiptera: Aphididae) following exposure to LC_5_ and LC_15_ of acetamiprid and imidacloprid (Ullah et al. 2019a; 2019b). The sublethal concentrations of nitenpyram, pirimicarb, and flonicamid enhanced fecundity in *A. gossypii* (Koo et al. 2015; Wang et al. 2017). Similar effects were noted for *Myzus persicae* (Sulzer) (Hemiptera: Aphididae) when treated to the LC_25_ of flupyradifurone (Tang et al. 2019). In addition to other effects, sublethal concentrations of insecticides may interfere with the feeding behavior of target insect pests (Miao et al. 2014; Zeng et al. 2016; Yuan et al. 2017).

The age-stage, two-sex life table is used for studying the lethal and intergenerational, transgenerational or multi-generational sublethal effects of insecticides on insects (e.g., Gul et al. 2021; 2023; Chi et al. 2020; 2023a). In contrast to the traditional female age-specific life table analysis, this approach accounts for stage differentiation and provides more accurate estimates of various life table parameters (Chi et al. 2020; Ding et al. 2021; Chi et al. 2022a; 2022b). Additionally, digital monitoring of insect feeding by the Electrical Penetration Graph (EPG) system is a promising tool (Tariq et al. 2017; Yuan et al. 2017; Milenovic et al. 2019). The EPG technique has been widely used for the detailed investigation of the feeding behavior of piercing-sucking insects. To determine a suitable plant for feeding, aphid do several probing attempts (Sauge et al. 2002). Cho et al. (2011) and Gul et al. (2023) reported that the sublethal concentrations of flonicamid and thiamethoxam affect the feeding behavior of aphids. The LC_30_ of flonicamid and imidacloprid significantly decreased the total duration of phloem ingestion of *A. gossypii* (Koo et al. 2015). Miao et al. (2014) showed that the LC_10_ and LC_50_ of imidacloprid, dinotefuran, thiacloprid and thiamethoxam cause a higher percentage of no probing phase and shorter phloem sap ingestion phase on treated wheat plants. These studies showed that the sublethal concentrations of insecticides significantly affect the feeding behavior of targeted insects.

In this study, we determined the toxicity of thiamethoxam against *S. graminum* and calculate the LC_5_ and LC_10_ concentrations. We used these concentrations to investigate the hormetic effects of thiamethoxam on survival, development, fecundity, and population projection of *S. graminum* using an age-stage, two-sex life table approach. Furthermore, the feeding behavior of *S. graminum* has also been investigated following exposure to the LC_5_ and LC_10_ concentrations of thiamethoxam.

## Material and methods

### Study insect

The apterous *S. graminum*, originally collected from the wheat field, was reared for more than two years at the National Agricultural Research Center (NARC), Islamabad, Pakistan. The parthenogenetic colony of *S. graminum* was maintained on wheat seedlings without any insecticide exposure under laboratory conditions with a temperature of 18 ± 2 °C, 60 ± 5% RH, and a photoperiod of 16:8 L: D.

### Bioassays

The thiamethoxam (Actara^®^ 25 WG) insecticide was provided by Syngenta Pakistan Ltd. To determine the toxicity, thiamethoxam was diluted into five test concentrations (40, 20, 10, 5, and 2.5 mg L^−1^) from the corresponding stock solution. All serial concentrations were applied in bioassays immediately after preparation. The wheat plants at the leaf stage were sprayed with five concentrations separately using hand sprayers until run-off (adaxial and abaxial leaf sides). In the control group, the plants were sprayed with distilled water. The treated wheat plants were kept to dry at room temperature. Each insecticide concentration and control has three replicates, and thirty adult apterous aphids were used per replicate. The treated wheat plants containing aphids were kept under laboratory conditions with a temperature of 18 ± 2 °C, 60 ± 5% RH, and a photoperiod of 16:8 L: D. The mortality was recorded at 48 h after exposure to thiamethoxam. Aphids not moving when pushed gently with a soft brush were considered dead.

### Sublethal effects of thiamethoxam on *Schizaphis graminum* (F_0_)

We employed the sublethal concentrations (LC_5_ and LC_10_) of thiamethoxam to elucidate their impact on directly exposed *S. graminum* (F_0_) after likely occurring due to insecticide degradation under field conditions. The healthy wheat plants were sprayed with the LC_5_ (2.259 mg L^−1^) and LC_10_ (3.057 mg L^−1^) values calculated by a log-probit model and determined using the previously described bioassay using a hand sprayer until run-off (adaxial and abaxial leaf sides). In the control treatment, the wheat plants were sprayed with distilled water. The wheat plants that had been treated were allowed to dry at room temperature. Apterous adult aphids were transferred to the insecticide-treated and control wheat plants. The treated wheat plants were kept under laboratory conditions with a temperature of 18 ± 2 °C, 60 ± 5%, and a photoperiod of 16:8 L:D. After 48 h treatment, forty survived, and healthy aphids were individually transferred to micro cages containing insecticide-free wheat plants. Each aphid was considered a single replicate. The longevity and fecundity of *S. graminum* were recorded daily. After counting, the newly born nymphs were removed from the cage. The data were continuously recorded until the death of all aphids.

### Intergenerational impact of thiamethoxam on *Schizaphis graminum* (F_1_)

The intergenerational impact of LC_5_ and LC_10_ of thiamethoxam on the succeeding parthenogenetically F_1_ generation of *S. graminum* was checked following the same experimental setup. Forty newly-born nymphs from F_0_ parents - the F_1_ individuals - were randomly selected and transferred to clean micro cages containing insecticide-free fresh wheat plants individually. Each aphid was considered a single replicate. The survival and developmental duration of F_1_ aphids were recorded daily. The daily fecundity (nymphs per aphid) was counted and removed daily until death. The experiments were performed under standard laboratory conditions as described above.

### Electropenetrography of *Schizaphis graminum* feeding behavior

The feeding behavior of adult *S. graminum* on wheat plants treated with the LC_5_ and LC_10_ of thiamethoxam was monitored using an eight-channel DC-EPG (Wageningen University, The Netherlands). Moreover, we investigated the intergenerational effects on the feeding behavior of progeny generation adults whose parents were treated with the LC_5_ and LC_10_ of thiamethoxam. Briefly, the wheat plants were sprayed with the LC_5_ and LC_10_ using a hand sprayer until run-off (adaxial and abaxial leaf sides). Plants were sprayed with distilled water for blank controls. The treated plants were air-dried for 2 h at room temperature before experiments. Adult aphids were starved for approximately 1 h between wiring and the beginning of the EPG experiment. After starvation, aphids were individually connected via their dorsum to a gold wire (18 μm in diameter and 6–8 cm in length) using a small drop of high purity silver conductive paint. The insect attached to the gold wire was then carefully placed on the treated wheat plants. The gold wire was connected to Giga-8 DC-EPG amplifier with 10^9^Ω input resistance and an adjustable plant voltage. A copper wire (2 mm in diameter and 5 cm in length) which served as a plant electrode, was inserted into the pot soil to provide voltage. The waveforms were recorded simultaneously from eight plants with alternate channels of water or thiamethoxam-treated plants. To avoid external electrical noises, the experiments were conducted in an electrically earthed Faraday cage (2 × 2 × 4 feet, aluminum frame with a steel base) at 18 °C and 60–65% RH under continuous light for eight hours using PROBE 3.4 software (Wageningen Agricultural University, Wageningen, The Netherlands). Freshly treated wheat plants and aphids were used for each replication. EPGs for each treatment were recorded for 8 h, which were used for final data analysis.

The EPG recordings were analyzed using Stylet+ Software. The variables of EPG were processed using EPG-Excel Data Workbook according to EPG ParProc. The EPG waveforms correlated with the probing activity were described as Np: Total duration of non-probing, Pr: Mean duration of probing, C: Total duration of intercellular stylet pathway, G: Total duration of xylem ingestion, E1: Total duration of salivary secretion into the sieve element, E2: Total duration of phloem sap ingestion and concurrent salivation.

### Data analysis

The LC_5_, LC_10_, and LC_50_ of thiamethoxam were calculated using log-probit model in PoloPlus 2.0 (LeOra Software Inc., Berkeley, CA). The electropenetrography (EPG) data were statistically analyzed using a one-way analysis of variance with Tukey’s post hoc test (IBM, SPSS Statistics, version 22).

### Life table data analysis

The raw data of control, LC_5,_ and LC_10_ treated F_0_ cohorts and their progeny (F_1_) were analyzed using the age-stage, two-sex life table method (Chi, 1988; Chi and Liu, 1985; Chi et al. 2020; Chi et al. 2023a). The development time, female longevities, reproductive days (RP_*d*_), adult pre-reproductive period (APRP), total pre-reproductive period (TPRP), and fecundity (*F*) (nymphs/female), as well as the demographic traits including the intrinsic rate of increase (*r*), finite rate of increase (*λ*), net reproductive rate (*R*_0_), and mean generation time (*T*) were determined using TWOSEX-MSChart computer program (Chi, 2023b; Chi et al. 2022a, 2022b). The standard errors were calculated by 100,000 bootstrap replicates (Huang and Chi. 2012; Amir-Maafi et al. 2022). The differences between the demographic parameters of control, LC_5_, and LC_10_ treated groups were determined using the paired bootstrap test at 5% significance level based on the confidence interval of difference (Wei et al. 2020). Details of the life table analysis were given as supplementary file.

### Population projection

Projections were made using the TIMING- MSChart program (Chi 2023c) based on the method of Chi and Liu. (1985) and Chi (1990). The population projection of *S. graminum* began with 10 newborn nymphs for each concentration, including the control under the assumption of no suppression by biotic and abiotic factors. It was projected for 50 days, a duration typically allowing *S. graminum* to establish and cause economically significant damage in winter wheat production. We conducted a comprehensive analysis using 100,000 bootstrap results of the finite rate of increase (λ). Within this dataset, we identified the 2.5th and 97.5th percentiles, which corresponded to the 2500th and 97,500th sorted bootstrap samples, respectively. Subsequently, we utilized the life table samples from the bootstrap analysis that generated the 2.5th and 97.5th percentiles of the finite rate of increase (λ) to simulate the population’s growth over a 50-day period. This process allowed us to assess and visualize the variability and uncertainty in the projected populations, providing valuable insights into the confidence intervals associated with our results (Huang et al. 2017).

## Results

### Toxicity of thiamethoxam on *Schizaphis graminum*

The toxicity results of thiamethoxam against *S. graminum* adults after exposure for 48 h showed that the LC_50_ value was 8.89 mg L^−1^ (95% confidence interval [CI] 7.811–10.077 mg L^−1^). The LC_5_ and LC_10_ values were found as 2.259 mg L^−1^ (95% CI: 1.657–2.851 mg L^−1^) and 3.057 mg L^−1^ (95% CI 2.360–3.725 mg L^−1^), respectively (Table 1).

**Table 1:** Toxicity of thiamethoxam against adult *Schizaphis graminum* after 48 h exposure

*n*^a^	Slope ± SE^b^	LC_5_ mgL^−1^ (95% CI)^c^	LC_10_ mgL^−1^ (95% CI)^c^	LC_50_ mgL^−1^ (95% CI)^c^	*χ*^2^ (*df*)^d^	*P*-value
540	2.764 ± 0.228	2.259 (1.657–2.851)	3.057 (2.360–3.725)	8.89 (7.811–10.077)	5.998 (13)	0.946

^a^Number of insects

^b^Standard error

^c^95% confidence intervals

^d^Chi-square value (*χ*^2^) and degrees of freedom (*df*) as calculated by PoloPlus 2.0

### Impact of LC_5_ and LC_10_ of thiamethoxam on parental aphids (F_0_)

The 48-h (48 h) LC_5_ and LC_10_ of thiamethoxam significantly affected the longevity and fecundity of adult *S. graminum* (Table 2). The longevity and fecundity of *S. graminum* significantly decreased following exposure to the LC_5_ and LC_10_ of thiamethoxam as compared to control (*P* < 0.05). The number of the reproductive days were lowest on LC_10_ of thiamethoxam treated individuals (Table 2, *P* < 0.05).

**Table 2:** Adult longevity, fecundity and reproductive days of the control, LC5 and LC10 of thiamethoxam treated F_0_ generation of *Schizaphis graminum*

Parameters	Control (Mean ± SE)	LC_5_ (Mean ± SE)	LC_10_ (Mean ± SE)
Adult Longevity (days)	19.13 ± 0.74 a	16.13 ± 0.69 b	12.08 ± 0.60 c
Fecundity (nymphs/female)	39.18 ± 2.08 a	29.28 ± 1.54 b	26.13 ± 1.68 b
Reproductive days (days)	14.93 ± 0.69 a	11.18 ± 0.51 b	9.63 ± 0.56 c

Standard errors were estimated by using the bootstrap technique with 100,000 resampling. Difference was compared using the paired bootstrap test (*P* < 0.05). The means within a row followed by a different lowercase letters indicate significant differences among the treatments

### Developmental duration and adult longevity of F_1_*Schizaphis graminum*

The intergenerational sublethal effects on F_1_ *S. graminum* whose parents (F_0_) were treated with the LC_5_ and LC_10_ of thiamethoxam are shown in Table 3. Results showed that the developmental period of 1st instar significantly decreased (*P* < 0.05) with the LC_5_ of thiamethoxam, while no effects were observed for the LC_10_ group as compared to control (Table 3). The developmental duration of 3rd instar *S. graminum* was significantly reduced (*P* < 0.05) at LC_5_ concentration compared to control. However, no effects were noted at the LC_10_ (*P* > 0.05). Similarly, the 4th instar duration was also decreased (*P* < 0.05) in LC_5_ treated group as compared to LC_10_ and control groups (Table 3). The duration of 2nd instar aphids was not affected at both concentrations (*P* > 0.05). Correspondingly, the pre-adult period of F_1_ *S. graminum* was significantly decreased (*P* < 0.05) when the parental aphids were exposed to the LC_5_ of thiamethoxam compared to LC_10_ and control groups. In contrast, the adult longevity of F_1_ aphids was significantly increased (*P* < 0.05) at the LC_5_, while no effects were observed at LC_10_ as compared to control (Table 3). The age-stage specific survival rate (*s*_*xj*_) shows the probability that a newly born nymph of *S. graminum* will survive to age *x* and stage *j* (Fig. S1). Various overlaps were observed among the LC_5_, LC_10_, and control due to the differences in the developmental and adult stages of *S. graminum*.

**Table 3:** Duration (days) of different developmental stages of the control and F_1_ generation of *Schizaphis graminum*, whose parents (F_0_) were treated with LC_5_ and LC_10_ of thiamethoxam

Stage	Control (Mean ± SE)	LC_5_ (Mean ± SE)	LC_10_ (Mean ± SE)
First-instar nymph	1.73 ± 0.09 a	1.33 ± 0.10 b	1.63 ± 0.10 a
Second-instar nymph	1.53 ± 0.08 a	1.38 ± 0.10 a	1.43 ± 0.11 a
Third-instar nymph	1.93 ± 0.10 a	1.45 ± 0.11 b	1.85 ± 0.12 a
Fourth-instar nymph	1.83 ± 0.09 a	1.38 ± 0.10 b	1.90 ± 0.12 a
Pre-adult	7.00 ± 0.12 a	5.53 ± 0.11 b	6.80 ± 0.13 a
Adult (Female)	21.13 ± 0.85 b	24.58 ± 0.87 a	21.15 ± 0.84 b

Standard errors were estimated by using the bootstrap technique with 100,000 resampling. Difference was compared using the paired bootstrap test (*P* < 0.05). The means within a row followed by a different lowercase letters indicate significant differences among the treatments

### Fecundity and life table parameters of F_1_*Schizaphis graminum*

**Fig. 1:**
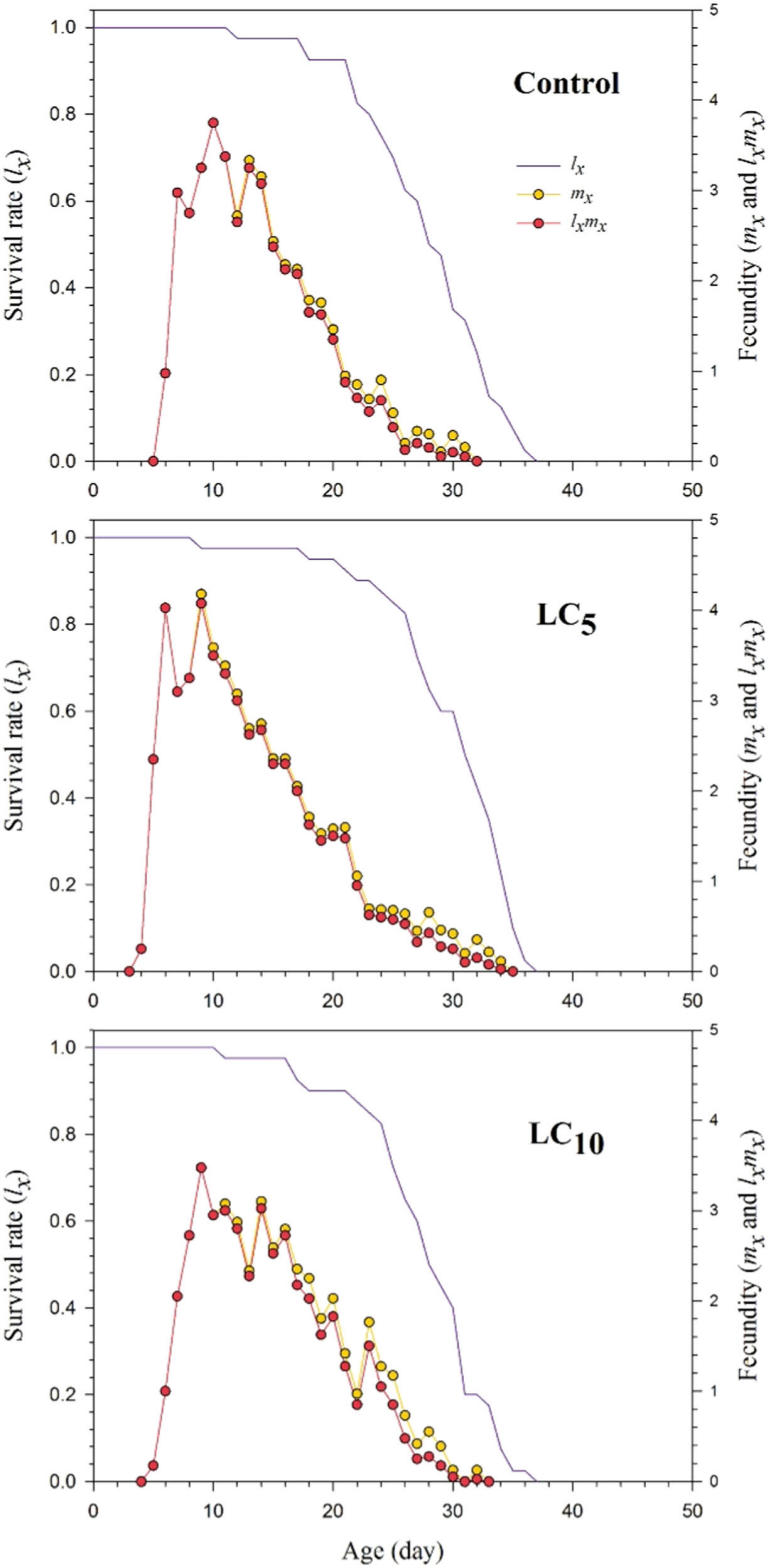
Population age-specific survival rate (*l*_*x*_), age-specific fecundity (*m*_*x*_) and the age-specific maternity (*l*_*x*_*m*_*x*_) for F_1_ generation *Schizaphis graminum* descending from F_0_ individuals treated with the LC_5_ and LC_10_ of thiamethoxam and control

The age-specific survival rate (*l*_*x*_), age-specific fecundity (*m*_*x*_) and the age-specific maternity (*l*_*x*_*m*_*x*_) curves for the LC_5_, LC_10_, and control groups were presented in Fig. 1. The *l*_*x*_, *m*_*x*_ and *l*_*x*_*m*_*x*_ parameters were affected in the LC_10_ while stimulated in the LC_5_ of thiamethoxam as compared to control. The age-stage specific survival rate (*e*_*xj*_) shows the expected duration of an individual aphid of age *x* and stage *j* that will survive after the age *x* (Fig. S2). The curves represent that the F_1_ generation of *S. graminum* is expected to live longer in the LC_5_ treatment while shorter in the LC_10_ of thiamethoxam as compared to control. The age-stage reproductive value (*v*_*xj*_) curves show the as the contribution of individuals of age *x* and stage *j* to the future population (Fig. S3). The maximum *v*_*xj*_ values were noted in the LC_5_ treated insects, whereas the minimum values were observed in LC_10_ of thiamethoxam as compared to the control.

The intergenerational impact of thiamethoxam on the reproduction and life table parameters of F_1_ aphids whose parents were treated with the LC_5_ and LC_10_ concentrations are shown in Table 4. The results indicated that the net reproductive rate (*R*_0_) of F1 aphids at LC_5_ was 1.2 times higher than that of the control (*P* < 0.05), whereas no statistically significant difference was observed at the LC_10_ compared to the control (*P* > 0.05). Similarly, the *r* and *λ* were significantly increased (*P* < 0.05) in F_1_ individuals at the LC_5_ treatment compared to the control. However, no significant effects (*P* > 0.05) were noted in the LC_10_ treatment (Table 4). The *T* value was dramatically decreased (*P* < 0.05) at the LC_5_ of thiamethoxam compared to the LC_10_ and control groups. The fecundity (*F*) of F_1_ aphids was substantially enhanced (*P* < 0.05) only at the LC_5_ of thiamethoxam, while the reproductive days (RP_*d*_) were dramatically increased at both concentrations as compared to control (*P* < 0.05). Compared to control, the adult pre-reproductive period (APRP) and total pre-reproductive period (TPRP) were significantly reduced (*P* < 0.05) at the LC_5_, while no effects were observed at the LC_10_ of thiamethoxam (Table 4).

**Table 4:** Reproduction and life table parameters of control and F1 generation of *Schizaphis graminum*, whose parents (F_0_) were treated with LC_5_ and LC_10_ of thiamethoxam

Parameters	Control (Mean ± SE)	LC_5_ (Mean ± SE)	LC_10_ (Mean ± SE)
*R*_0_ (offspring/individual)	41.10 ± 1.60 b	49.70 ± 2.33 a	43.15 ± 1.98 b
*r* (day^−1^)	0.3146 ± 0.0057 b	0.3861 ± 0.0083 a	0.3071 ± 0.0061 b
*λ* (day^−1^)	1.3698 ± 0.0079 b	1.4712 ± 0.0121 a	1.3595 ± 0.0083 b
*T* (days)	11.81 ± 0.19 a	10.12 ± 0.19 b	12.26 ± 0.24 a
*F* (nymphs/female)	41.10 ± 1.60 b	49.70 ± 2.33 a	43.15 ± 1.98 b
RP_*d*_ (days)	13.78 ± 0.56 b	17.05 ± 0.70 a	15.23 ± 0.69 ab
APRP (days)	0.50 ± 0.14 a	0.10 ± 0.05 b	0.38 ± 0.10 a
TPRP (days)	7.50 ± 0.18 a	5.63 ± 0.12 b	7.18 ± 0.17 a

*R*_0_ net reproductive rate, *r* intrinsic rate of increase, *λ* finite rate of increase, *T* mean generation time, *F* fecundity, RP_*d*_ reproductive days, *APRP* adult prereproductive period, *TPRP* total prereproductive period

Standard errors were estimated by using the bootstrap technique with 100,000 resampling. Difference was compared using the paired bootstrap test (*P* < 0.05). The means within a row followed by a different lowercase letters indicate significant differences among the treatments

### Population projection

**Fig. 2:**
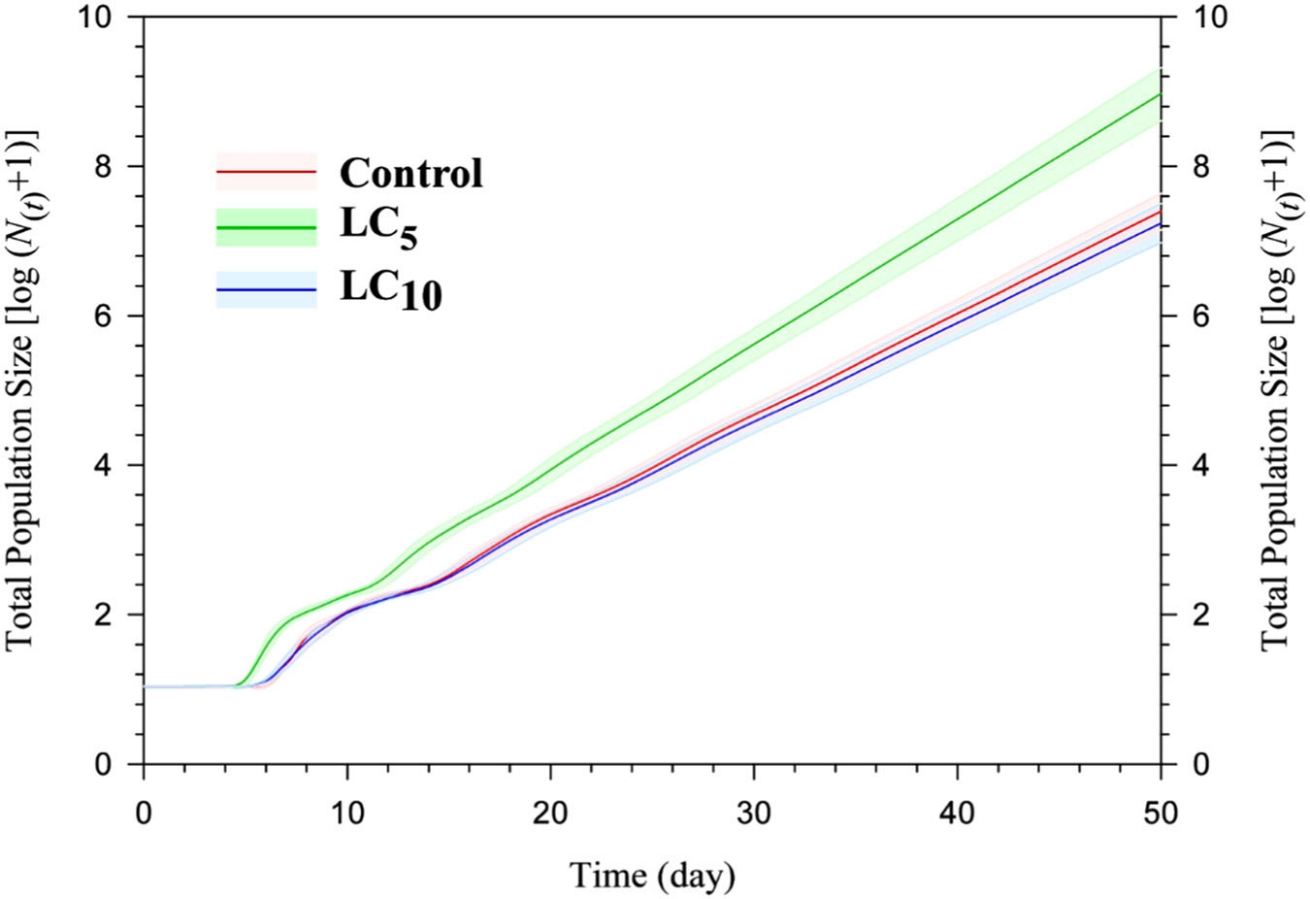
Total population size (*N*_*t*_) after projection of control and F_1_ progeny of *Schizaphis graminum* produced by LC_5_ and LC_10_ of thiamethoxam treated parents and control for a 50-day period by using life table data. (All data are in log base 10 and the shaded areas represent the limits of the 95% CIs based on the 2.5 and 97.5% percentiles of λ, finite rate of increase)

The original, 2.5th, and 97.5th percentiles of population projections of the F_1_ progeny of *S. graminum* produced by LC_5_ and LC_10_ concentrations of thiamethoxam treated populations and control group are plotted in Fig. 2. The highest total population size was found in the population produced from LC_5_ of thiamethoxam and was projected to surpass 9.0 × 10^8^ individuals after 50 days. The population produced from LC_10_ of thiamethoxam treated *S. graminum* yielded the lowest population size estimate with ~1.7 × 10^7^, while the control group was ~2.4 × 10^7^ after 50 days.

### Sublethal effects of thiamethoxam on feeding behavior of F_0_ and F_1_*S. graminum*

**Fig. 3:**
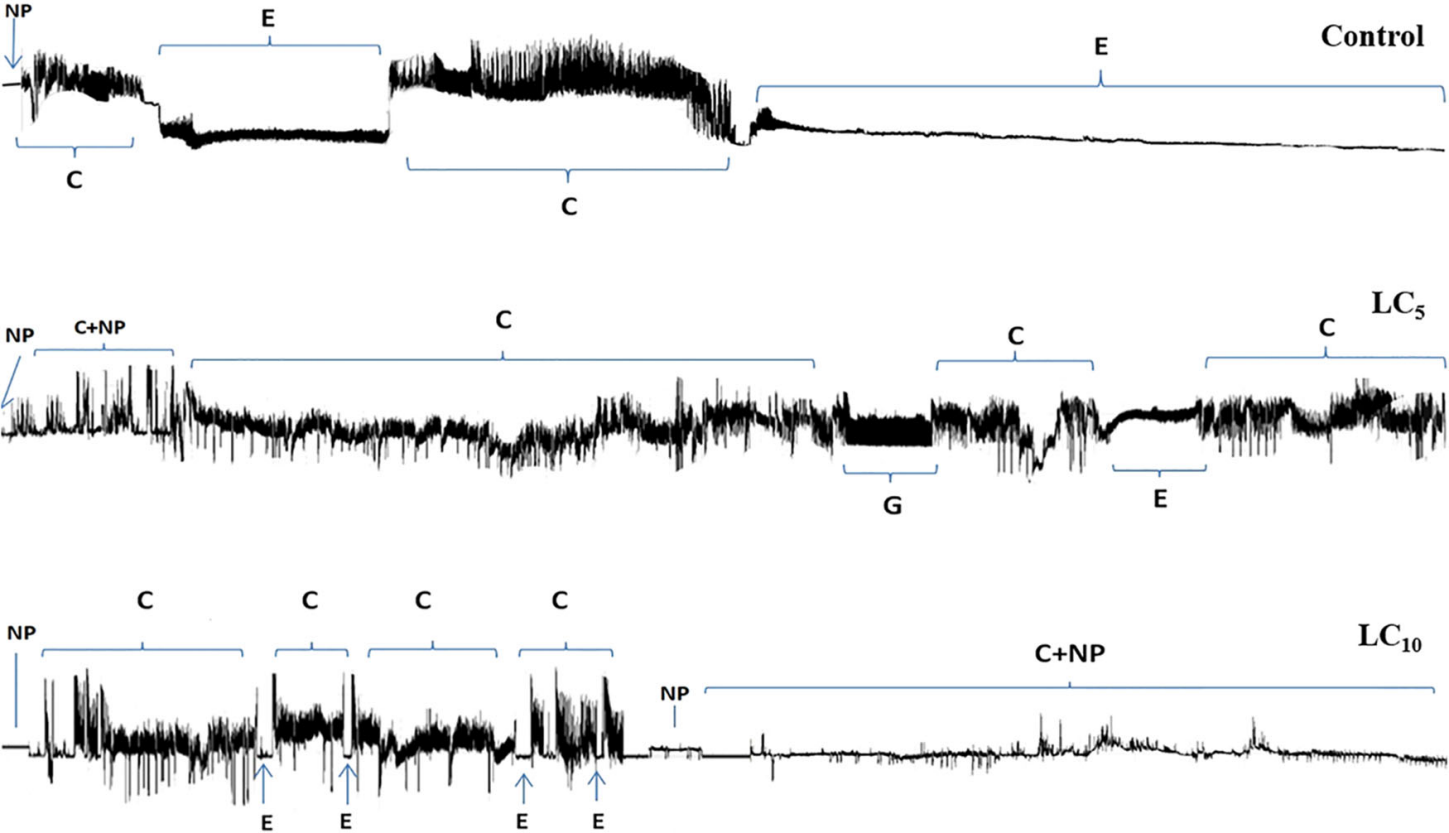
EPG waveforms recorded from *Schizaphis graminum* on wheat plants treated with the LC_5_ and LC_10_ of thiamethoxam and control. The EPG waveforms were described as Np: Total duration of non-probing, Pr: Mean duration of probing, C: Total duration of intercellular stylet pathway, G: Total duration of xylem ingestion, E1: Total duration of salivary secretion into the sieve element, E2: Total duration of phloem sap ingestion and concurrent salivation

The LC_5_ and LC_10_ of thiamethoxam significantly affected the feeding behavior of directly exposed adult *S. graminum* as compared to the control insects (Table 5). The waveforms recorded from *S. graminum* on wheat plants treated with the LC_5_ and LC_10_ of thiamethoxam are shown in Figs. 3, S4. Results showed that the Np durations (total duration of non-probing) of the LC_5_ and LC_10_ treatments were 1610 and 1305 s, respectively, which were significantly (*P* < 0.05) longer than the control group (460 s). The mean duration of Pr (mean duration of probing) was substantially decreased in the LC_5_ (19842 s) and LC_10_ (19211 s) concentrations of thiamethoxam as compared to control (20984 s) (*P* < 0.05). The total duration of C (total duration of intercellular stylet pathway) was 7653 s in LC_5_ and 8323 s in LC_10_ while the total duration of E1 (total duration of salivary secretion into the sieve element) was 1465 s in LC_5_ and 1238 s in LC_10_ treatments, which are significantly (*P* > 0.05) longer than control aphids (Table 5). Moreover, the total duration of E2 (total duration of phloem sap ingestion and concurrent salivation) substantially (*P* > 0.05) increased in the LC_5_ (9665 s) and LC_10_ (7134 s) treatments as compared to control (14969). No significant differences (*P* > 0.05) were observed in total duration of G (total duration of xylem ingestion) among the thiamethoxam treated insects and control (Table 5).

**Table 5:** Sublethal effects of thiamethoxam on the probing and feeding behavior of *Schizaphis graminum* on wheat plants treated with the LC_5_ and LC_10_ concentrations and control

Treatments	Np	Pr	C	G	E1	E2
Control	460.9 ± 86.3 b	20984 ± 101.7 a	3665.6 ± 729.2 b	1894.3 ± 998.5 a	241 ± 44.7 b	14969 ± 1429.3 a
LC_5_	1610.5 ± 305 a	19842 ± 286.1 b	7653.2 ± 680.5 a	927 ± 383.6 a	1465 ± 268.5 a	9665 ± 626.1 b
LC_10_	1305.2 ± 166 a	19211 ± 370.5 b	8323.5 ± 945.6 a	400 ± 262.3 a	1238 ± 261.5 a	7134 ± 587.7 b

The EPG parameters are: Np: Total duration of non-probing, Pr: Mean duration of probing, C: Total duration of intercellular stylet pathway; G: Total duration of xylem ingestion; E1: Total duration of salivary secretion into the sieve element; E2: Total duration of phloem sap ingestion and concurrent salivation. Data represent means ± SEM. Different lowercase letters within the same column represent significant differences at *P* < 0.05 level (one-way ANOVA followed by Tukey’s post hoc test)

**Fig. 4:**
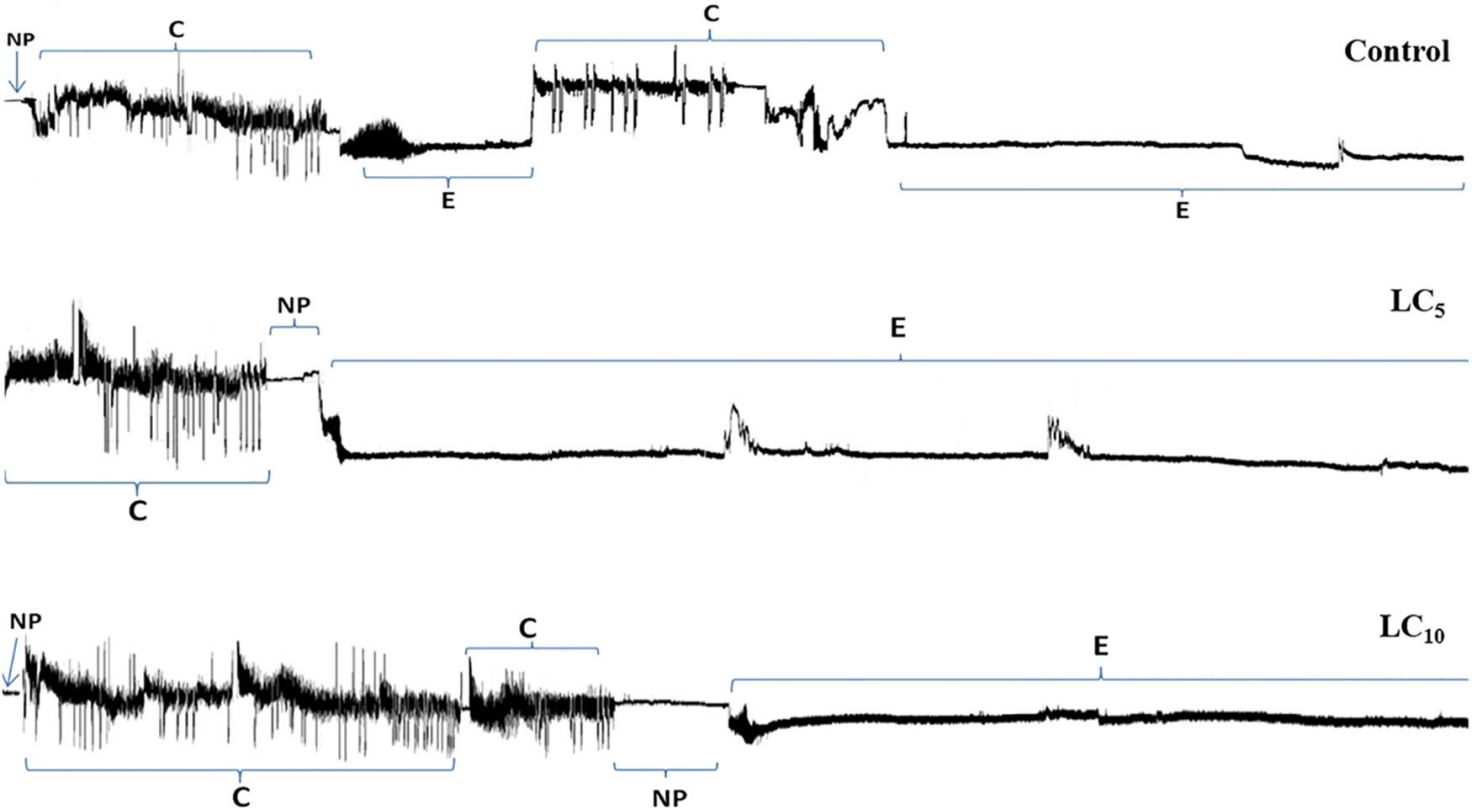
EPG waveforms recorded from progeny generation *Schizaphis graminum* (F1) descending from parental aphids treated with the LC_5_ and LC_10_ of thiamethoxam. The EPG waveforms were described as Np: Total duration of non-probing, Pr: Mean duration of probing, C: Total duration of intercellular stylet pathway, G: Total duration of xylem ingestion, E1: Total duration of salivary secretion into the sieve element, E2: Total duration of phloem sap ingestion and concurrent salivation

The intergenerational sublethal effects on the probing and feeding behavior were checked on F_1_ adult aphids whose parents (F_0_) were treated with the LC_5_ and LC_10_ of thiamethoxam (Table 6). The waveforms recorded from progeny generation *S. graminum* (F_1_) descending from parental aphids treated with the LC_5_ and LC_10_ of thiamethoxam are shown in Fig. 4. Results showed that the Np duration of F_1_ individuals was 463.8 sec in the LC_5_ treated group, which is significantly shorter than the LC_10_ (985.7 s) and control groups (1000.2 s) (*P* < 0.05). The total duration of Pr was substantially longer (*P* < 0.05) in the LC_5_ treatment (21028 s) as compared to LC_10_ (19945 s) and control (20419 s). Furthermore, the total duration of E2 in the LC_5_ treated group was 18,452 s which was significantly (*P* < 0.05) longer than the LC_10_ (14,162 s) and control aphids (14,222 s) (Table 6). The total duration of C, G and E1 were statistically same among the thiamethoxam treated aphids and control (*P* > 0.05) (Table 6).

**Table 6:** Intergenerational impact of thiamethoxam on the probing and feeding behavior of *Schizaphis graminum* (F_1_) whose parents were treated with the LC_5_ and LC_10_ concentrations and control

Treatments	Np	Pr	C	G	E1	E2
Control	1000.2 ± 285.28 a	20419 ± 316.9 ab	3857.8 ± 880.41 a	977.7 ± 658.26 a	416.02 ± 211.98 a	14222 ± 1070.99 b
LC_5_	463.8 ± 178.01 b	21028 ± 164.1 a	2191.3 ± 218.57 a	125.5 ± 125.46 a	258.82 ± 118.20 a	18452 ± 544.26 a
LC_10_	985.7 ± 275.62 a	19945 ± 266.7 b	3701.5 ± 735.42 a	1829.9 ± 921.83 a	451.66 ± 182.51 a	14162 ± 1118.43 b

The EPG parameters are: Np: Total duration of non-probing; Pr: Mean duration of probing; C: Total duration of intercellular stylet pathway; G: Total duration of xylem ingestion; E1: Total duration of salivary secretion into the sieve element; E2: Total duration of phloem sap ingestion and concurrent salivation. Data represent means ± SEM. Different letters within the same column represent significant differences at *P* < 0.05 level (one-way ANOVA followed by Tukey’s post hoc test)

## Discussion

In this study, we investigated the sublethal and intergenerational effects of thiamethoxam on two consecutive generations (F_0_ and F_1_) of *S. graminum*. The results demonstrated that thiamethoxam displayed high toxicity against *S. graminum*, with an LC_50_ of 8.89 mg/l following 48-h treatment. In addition to its lethal effects, thiamethoxam induces intergenerational sublethal and hormetic effects on the biological parameters of the exposed *S. graminum*. Similar effects have also been recorded in *A. gossypii* (Ullah et al. 2020). These results suggested that the LC_5_ and LC_10_ concentrations may be critical for managing *S. graminum* in field conditions.

The current study shows that the longevity and fecundity of adult *S. graminum* (F_0_) were reduced following exposure to the LC_5_ and LC_10_ of thiamethoxam for 48 h. Our results align with Ma et al. (2022) who demonstrated that the longevity and fecundity of parental adult *A. gossypii* (F_0_ generation) significantly declined when treated with LC_10_ of afidopyropen. Likewise, Ullah et al. (2019a) reported decreased longevity and fecundity of *A. gossypii* when directly exposed to the LC_5_ and LC_15_ of imidacloprid. The negative consequences, such as shorter lifespan and decreased fertility, were also observed in *M. persicae* when treated with flupyradifurone at sublethal concentrations (Tang et al. 2019). The total longevity and fecundity of *S. graminum* were significantly reduced following exposure to acetamiprid (Vakhide and Safavi. 2014). Cui et al. (2018) also reported decreased longevity and fecundity when parental *A. gossypii* (F_0_) were treated with the sublethal concentration of cycloxaprid. These results showed that along with lethal effects, the sublethal concentrations of insecticides greatly affect the adult lifespan and fertility of the surviving aphids. Therefore, it is crucial to investigate the sublethal effects of commonly used insecticides on target insects to better understand their efficacy even after degradation due to biotic and abiotic constraints.

On the other hand, the developmental stages of F_1_ *S. graminum* were positively affected when the parental aphids (F_0_) were exposed to the LC_5_ of thiamethoxam. Results showed that the developmental duration of 1st, 3rd, and 4th instar of F_1_ *S. graminum* was significantly decreased at the LC_5_ as compared to LC_10_ treated group and control. The pre-adult stage was significantly shorter in progeny aphids (F_1_) when parental generation (F_0_) was treated with the LC_5_ of thiamethoxam compared to LC_10_ and control groups. In contrast, the adult longevity of F_1_ *S. graminum* was substantially prolonged when the parental aphids (F_0_) were treated with the LC_5_, while no effects were observed for the LC_10_ as compared to control. These results indicated that the LC_5_ of thiamethoxam positively affects the development and overall lifespan of *S. graminum* following parental adults after 48 h exposure. Ullah et al. (2019a) reported decreased developmental duration of 4th instar and pre-adult stage of F_1_ *A. gossypii* when parental aphids (F_1_) were treated with the LC_5_ and LC_15_ of imidacloprid. The developmental duration of 3rd and 4th instars and pre-adult stages of *M. persicae* was significantly decreased following 48 h exposure to the LC_25_ of flupyradifurone (Tang et al. 2019). The LC_15_ of thiamethoxam significantly reduced the 4th instar duration of F_1_ *A. gossypii* (Ullah et al. 2020). Yuan et al. (2017) also reported that the sublethal concentrations of cycloxaprid significantly decreased the developmental duration of F_1_ generation *A. gossypii*. The adult longevity of progeny generation of *A. gossypii* (F_1_) was significantly prolonged when parental generation (F_0_) was exposed to the LC_5_ and LC_15_ of imidacloprid and thiamethoxam (Ullah et al. 2019a; 2020). Tang et al. (2019) reported that the longevity of F_1_ and F_2_ generations of *M. persicae* were significantly extended when the parental aphids (F_0_) were treated with the LC_25_ of flupyradifurone. The male and female longevity of *Nilaparvata lugens* (Stål) (Hemiptera: Delphacidae) was significantly increased following exposure to the LC_20_ of nitenpyram for over 6 successive generations (Gong et al. 2022). The results of the current study and previous findings suggested that the sublethal concentrations of insecticides speed-up the developmental stages as well as increased the total longevity of target insect pests that ultimately enhance and support the pest outbreak in the field and causes severe damage to crops.

In the present study, the intergenerational hormetic effects were observed in *S. graminum* after exposure of the parental aphids to the LC_5_ of thiamethoxam compared to the LC_10_ and untreated aphids. For example, the female fecundity (*F*) and reproductive days (RP_*d*_) were significantly increased, while the adult pre-reproductive period (APRP) and total pre-reproductive period (TPRP) were markedly decreased in the progeny generation (F_1_) of LC_5_ treated group as compared to the LC_10_ and control aphids. Consequently, the demographic parameters, i.e., intrinsic rate of increase (*r*), finite rate of increase (*λ*) and net reproductive rate (*R*_0_), were significantly increased in the LC_5_ treatment as compared to LC_10_ and control. These changes in the life history traits of *S. graminum* indicated that the intergenerational hormetic effects occurred after 48 h exposure of parental aphids to the LC_5_ of thiamethoxam. This hormetic response occurred in *S. graminum* without any fitness tradeoffs following exposure to the LC_5_ and LC_10_ of thiamethoxam. Concurrent hormetic responses of multiple traits have been reported in *A. gossypii* exposed to thiamethoxam, imidacloprid and acetamiprid (Ullah et al. 2019a; Ullah et al. 2019b; Ullah et al. 2020), *M. persicae* exposed to flupyradifurone, acetamiprid, and imidacloprid (Ayyanath et al. 2013; Tang et al. 2019; Sial et al. 2018). Gong et al. (2022) examined transgenerational hormesis in brown planthopper (*N. lugens)* after six generations of 96 h exposure to LC_20_ nitenpyram. The pre-adult developmental duration and *T* were significantly decreased in F-Sub6 strain of *N. lugens* when subjected to the LC_20_ of nitenpyram (Gong et al. 2022). A similar phenomenon was also observed in our current study; the pre-adult developmental duration and *T* of *S. graminum* were substantially shortened in the LC_5_ treated group compared to the control. No significant effects were noted for the LC_10_ concentration of thiamethoxam. The population size of *S. graminum* projected at 50 days post-exposure was larger in the LC_5_ treated group than in the LC_10_ and control groups. Priming hormesis may be necessary for agricultural insect pests likely to encounter multiple and successive low and sublethal stress levels (Rix et al. 2016; Cutler et al. 2022). Overall, the results of the present study strongly demonstrated that exposure to the LC_5_ and LC_10_ of thiamethoxam caused intergenerational hormetic effects on the demographic characteristics of *S. graminum*. This increased reproduction and longevity might causes the pest outbreak under field contexts which ultimately increase crop damage.

In addition to other parameters, we investigated the feeding behavior of parental and progeny *S. graminum* using electric penetration graph recordings (EPG) after exposure of F_0_ aphids to the LC_5_ and LC_10_ of thiamethoxam. Results showed that the total duration of non-probing (Np), total duration of intercellular stylet pathway (C), and total duration of salivary secretion into the sieve element were significantly increased, while mean duration of probing (Pr) and total duration of phloem sap ingestion and concurrent salivation (E2) were dramatically decreased in F_0_ adults following exposure to the LC_5_ and LC_10_ of thiamethoxam. These results demonstrated that aphids need more time to search appropriate nutritional sites when plants are exposed to the LC_5_ and LC_10_ of thiamethoxam. Similar results were shown by Miao et al. (2014) that the no probing phase was increased while the phloem sap ingestion phase was decreased in *Sitobion avenae* (Fabricius) (Hemiptera: Aphididae) on the wheat plants treated with LC_10_ and LC_50_ of thiamethoxam, imidacloprid, dinotefuran, and thiacloprid. Tariq et al. (2017) reported that inhibition of ingestion was dose-dependent, i.e., the increasing concentrations of flonicamid significantly enhanced the mean duration of non-probing phases while strongly inhibited the ingestion phases in cotton leafhopper, *Amrasca biguttula* Ishida (Hemiptera: Cicadellidae). The total duration of phloem sap ingestion and concurrent salivation (E2) were substantially reduced in F_0_ and F_1_ aphids after exposure to the sublethal concentrations of flonicamid (Gul et al. 2023). The LC_30_ of cyantraniliprole and imidacloprid significantly increased the total durations of intercellular stylet pathway (C) and mechanical probing difficulties (F) when green peach aphids feed on the treated tobacco plants (Zeng et al. 2016). The increasing concentrations of flonicamid and imidacloprid substantially increased the non-penetration phases (NP) and decreased the salivation (E1) and sap-feeding (E2) durations in *A. gossypii* (Koo et al. 2015). The LC_40_ of cycloxaprid had a negative impact on the phloem ingestion phases of *A. gossypii* (Yuan et al. 2017). The sublethal concentrations of cycloxaprid dramatically enhanced the non-probing durations and strongly inhibited the phloem ingestion phases of the treated *S. avenae* (Cui et al. 2012). All these results demonstrated that sublethal concentrations of insecticides negatively impact the survived sap-sucking insect pests. Interestingly, the total duration of Np was significantly decreased, while the total duration of E2 were significantly increased in the progeny generation (F_1_) following exposure of the parental aphids to the LC_5_ of thiamethoxam. Our results showed that the sublethal concentrations of thiamethoxam affect the feeding behavior of the directly exposed aphids (F_0_), while signifacantly increased the feeding behavior of the progeny generation. Here, we showed that the decreased longevity and fecundity of F_0_ aphids might be due to the direct effects of sublethal concentrations of insecticides on their feeding behavior, while enhanced reproduction and longevity may be due to the increased feeding behavior of F_1_ individuals that ultimately validated the hormetic effects. However, future studies are needed to investigate the in-depth mechanisms underlying the observed hormetic effects.

## Conclusion

Overall, our results show the LC_5_ and LC_10_ of thiamethoxam significantly affect the life span, fecundity, and feeding behavior of directly exposed F_0_ aphids. However, the LC_5_ concentration induces intergenerational hormetic effects on the biological parameters and feeding behavior of progeny generation (F_1_) of *S. graminum* that could increase crop damage. To the best of our knowledge, the present study is the only one determining thiamethoxam-induced intergenerational hormetic effects on the demographic parameters and feeding behavior of *S. graminum*. However, future studies should be conducted to investigate the multi-generational hormetic effects of thiamethoxam on *S. graminum* in field context.

### Supplementary Information

Supplementary data
