# Supplementary material for: Hormetic effects of thiamethoxam on Schizaphis graminum: demographics and feeding behavior
Source: Ecotoxicology. 2024 Mar 11;33(3):253–65. doi: 10.1007/s10646-024-02743-1 (PMC11009746; doi:10.1007/s10646-024-02743-1)
Supplement: Supplementary file 1 — Supplementary data [file 10646_2024_2743_MOESM1_ESM.docx]

*Supplementary data*

**Hormetic effects of thiamethoxam on Schizaphis graminum: demographics and feeding behavior**

Hina Gul ^1, 2^, Ihsan ul Haq ^2^, Farman Ullah ^3^, Shanza Khan ^2^, Aqsa Yaseen ^2^, Kaleem Tariq ^4^,

Ali Güncan ^5*^, Nicolas Desneux ^6^, Xiaoxia Liu ^1*^

^1^ MARA Key Laboratory of Pest Monitoring and Green Management, Department of Entomology, College of Plant Protection, China Agricultural University, Beijing 100193, China

^2^ Insect Pest Management Program, Institute of Plant and Environmental Protection, National Agricultural Research Centre, Islamabad, Pakistan

^3^ State Key Laboratory for Managing Biotic and Chemical Threats to the Quality and Safety of Agro-Products, Institute of Plant Protection and Microbiology, Zhejiang Academy of Agricultural Sciences, Hangzhou 310021, China

^4^ Department of Entomology, Abdul Wali Khan University Mardan, Khyber Pakhtunkhwa, Pakistan

^5^ Department of Plant Protection, Faculty of Agriculture, Ordu University, 52200, Ordu, Turkey

^6^ Université Côte d'Azur, INRAE, CNRS, UMR ISA, 06000 Nice, France

*Corresponding authors: [guncan.ali@gmail.com](mailto:guncan.ali@gmail.com) (A. Güncan), [liuxiaoxia611@cau.edu.cn](mailto:liuxiaoxia611@cau.edu.cn) (X. Liu);

**Life Table Analysis**

The age-specific survival rate (*l_x_*) and (*m_x_*) were determined using eq. 1 and 2:

 (1)

$m_{x}=\frac{\sum_{j=1}^{\beta} s_{xj}f_{xj}}{\sum_{j=1}^{k} s_{xj}}$ (2)

where *s_xj_* shows the probability of a newly born nymph that will survive to age *x* and stage *j*. *β* represents the number of stages, while *f_xj_* is the age-stage specific fecundity of the individual at age *x* and stage *j.*

The *RP_d_* represents number of days in which the females produce offspring and was calculated using eq. 3:

$RP_{d}=\frac{\sum_{x=1}^{N_{f}} D_{x}}{N_{f}}$ (3)

Where *N_f_* shows the number of female adults and *D_x_* represent the number of days that a female produced offspring ([Chen et al. 2018](#_ENREF_1)).

The intrinsic rate of increase (*r*) represents population growth rate when the time approaches infinity, and the population attains stable age-stage distribution. The *r* was estimated using the interactive bisection method and corrected with the Euler–Lotka equation with age indexed from 0 ([Goodman. 1982](#_ENREF_3)):

$\sum_{x=0}^{\infty} e^{-r\left(x+1\right)}l{}_{x}m_{x}=1$ (4)

The finite rate of increase (*λ*) indicates the population growth rate as the time approaches infinity and the population reaches the stable age stage distribution. The population size will increase at the rate of λ per time unit. The *λ* was calculated using eq. 5:

$\lambda=e^{r}$ (5)

The *R*_0_ shows the cumulative eggs laid by a single female till death. The *R*_0_ was estimated using eq. 6:

$R_{0}=\sum_{x=0}^{\infty} l_{x}m_{x}$ (6)

The *T* represents the time needed by a population to enhance to *R*_0_-fold its current size at a stable rate of increase. The *T* was estimated using eq. 7:

$T=\frac{\ln R_{0}}{r}$ (7)

The age-stage specific life expectancy (*e_xj_*) indicates the expected duration an individual of age *x* and stage *j* will survive. The *e_xj_* was estimated according to [Chi & Su (2006)](#_ENREF_2) using eq. 8:

$e_{xj}=\sum_{i=x}^{\infty} \sum_{y=j}^{\beta} s'_{iy}$ (8)

Where *s′_iy_* represents the possibility that an individual aphid of age *x* and stage *j* will survive to age *i* and stage *y* by assuming s′ = 1.

Age-stage specific reproductive value (*v_xj_*) represents the devotion to future offspring at age *x* and stage *j*. The *v_xj_* was estimated using eq. 9 according to ([Tuan et al. 2014](#_ENREF_4))

$v_{xj}=\frac{e^{r\left(x+1\right)}}{s_{xj}}\sum_{i=x}^{\infty} e^{-r\left(i+1\right)}\sum_{y=j}^{\beta} s'_{iy}f_{iy}$ (9)

**Supplementary figures:**

**
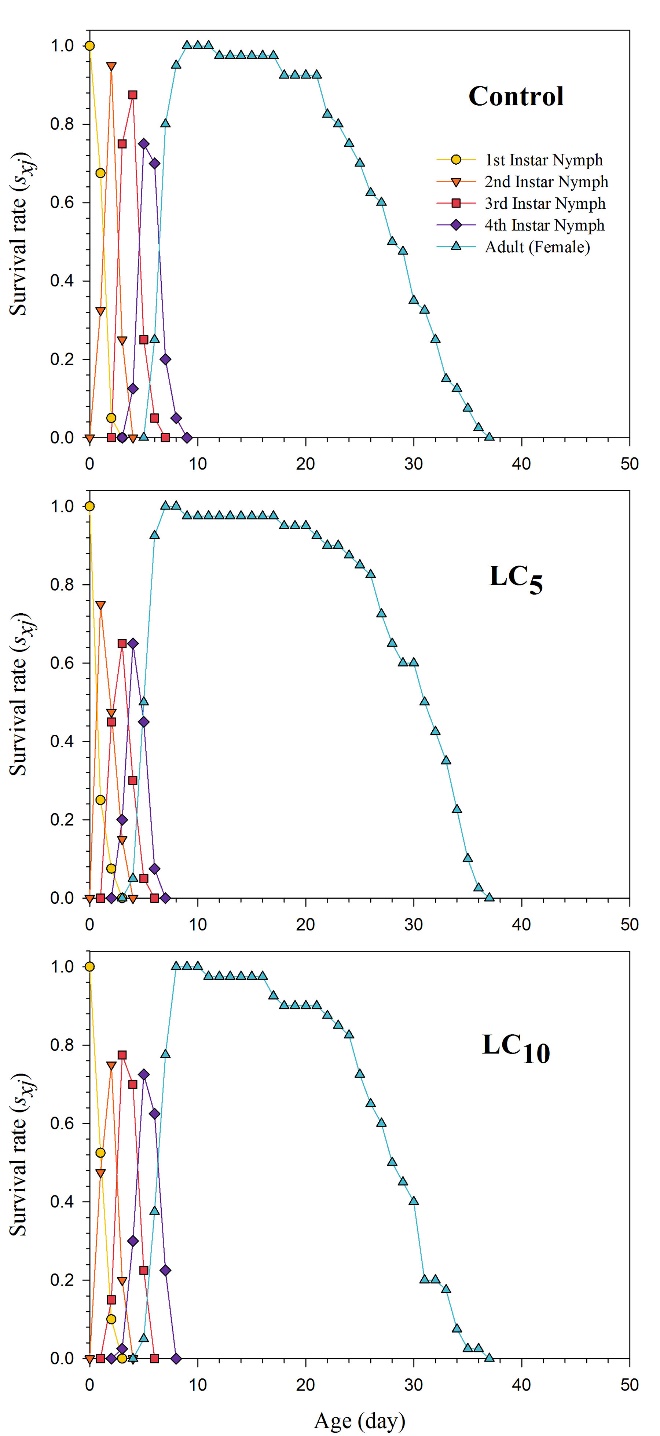
**

**Figure S1.** Age-stage specific survival rate (*s_xj_*) for F_1_ generation *Schizaphis graminum* descending from parents (F_0_) under control, treated with LC_5_, and LC_10_ of thiamethoxam.

**
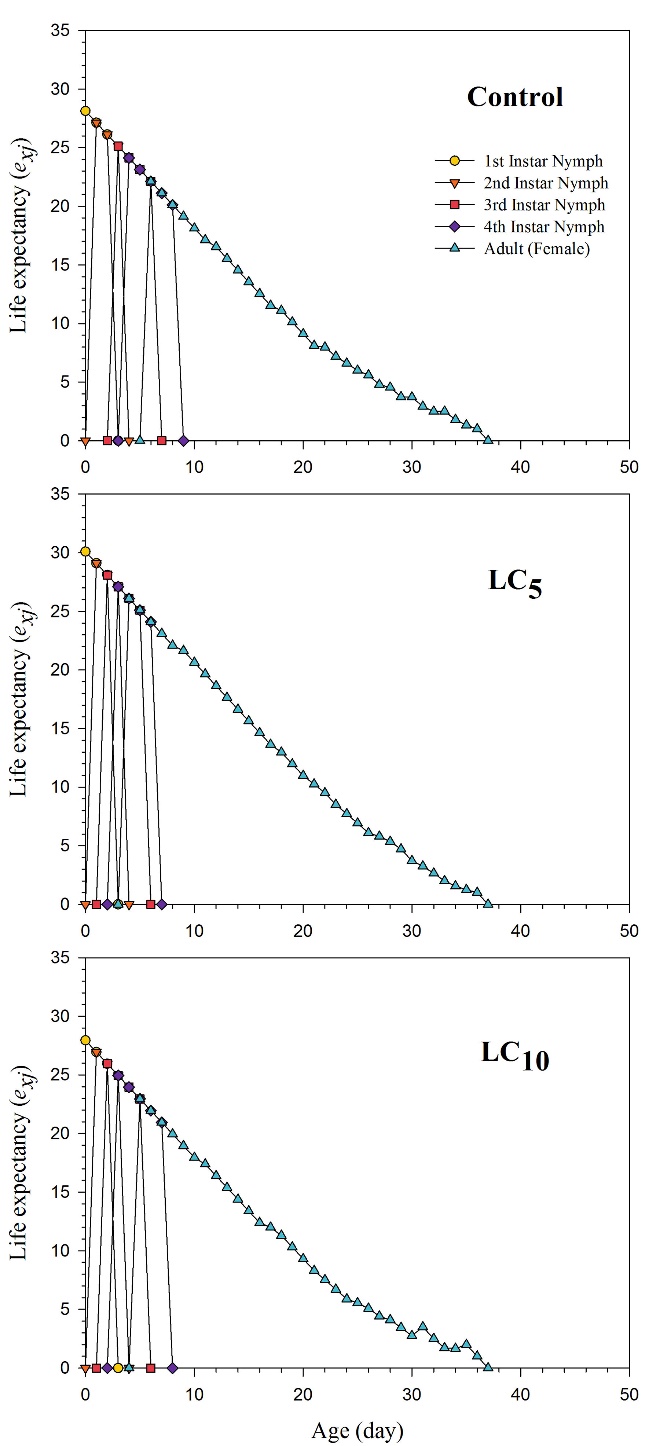
**

**Figure S2.** Age-stage specific survival rate (*e_xj_*) for F_1_ generation *Schizaphis graminum* descending from parents (F_0_) under control, treated with LC_5_, and LC_10_ of thiamethoxam.

**
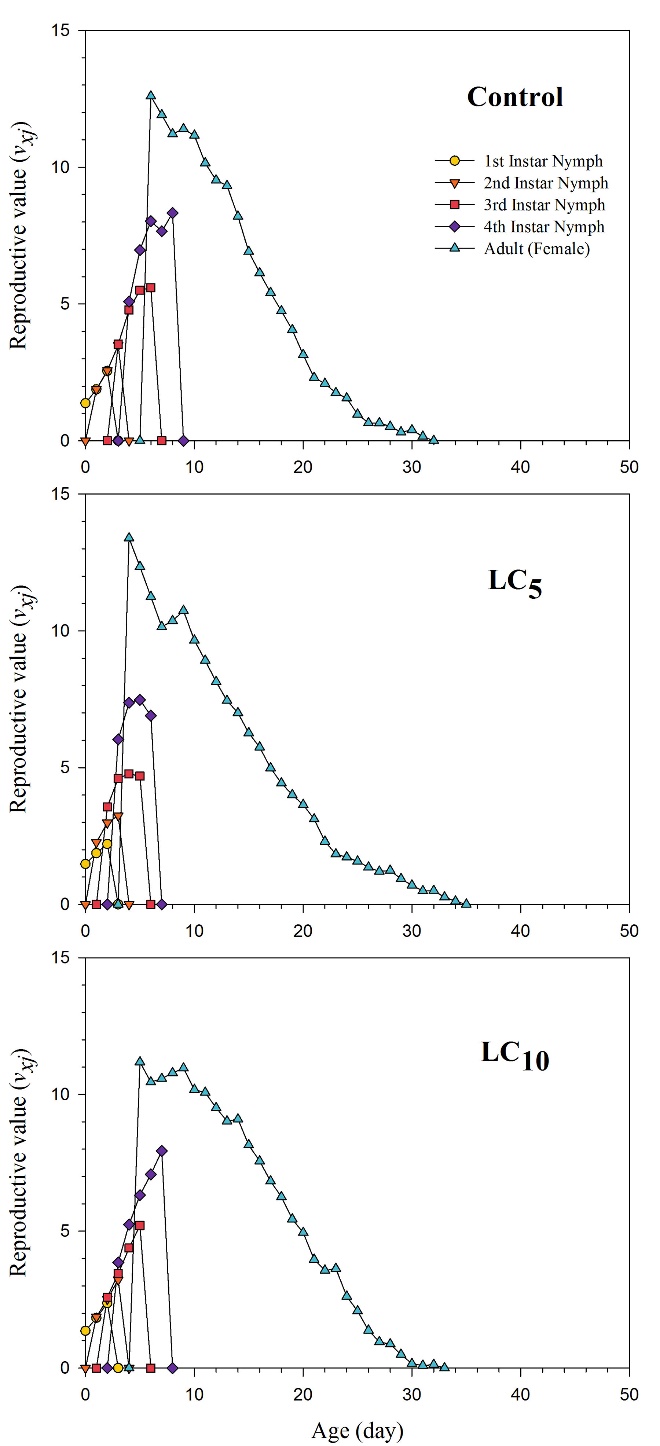
**

**Figure S3.** Age-stage reproductive value (*v_xj_*) for F_1_ generation *Schizaphis graminum* descending from parents (F_0_) under control, treated with LC_5_, and LC_10_ of thiamethoxam.

**
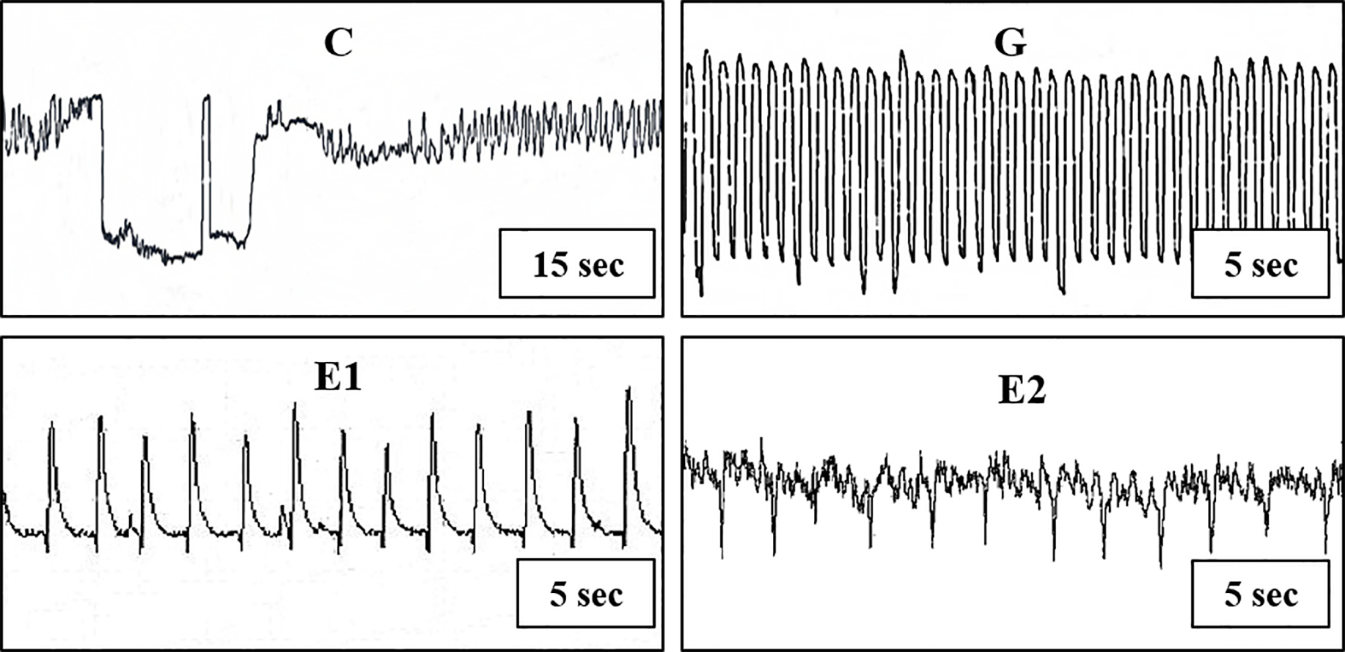
**

**Figure. S4.** Waveform C, G, E1, and E2 produced by adult *Schizaphis graminum* on wheat plants.

**References**

Chen, G-M., Chi, H., Wang, R-C., Wang, Y-P., Xu, Y-Y., Li, X-D., Yin, P & Zheng, F-Q (2018): Demography and uncertainty of population growth of *Conogethes punctiferalis* (Lepidoptera: Crambidae) reared on five host plants with discussion on some life history statistics. - Journal of Economic Entomology 111: 2143-2152.

Chi, H & Su, H-Y (2006): Age-stage, two-sex life tables of *Aphidius gifuensis* (Ashmead)(Hymenoptera: Braconidae) and its host *Myzus persicae* (Sulzer)(Homoptera: Aphididae) with mathematical proof of the relationship between female fecundity and the net reproductive rate. - Environmental Entomology 35: 10-21.

Goodman, D (1982): Optimal life histories, optimal notation, and the value of reproductive value. - The American Naturalist 119: 803-823.

Tuan, SJ., Lee, CC & Chi, H (2014): Population and damage projection of *Spodoptera litura* (F.) on peanuts (*Arachis hypogaea* L.) under different conditions using the age‐stage, two‐sex life table. - Pest Management Science 70: 805-813.
